# Early and intense epidemic of respiratory syncytial virus (RSV) in Denmark, August to December 2022

**DOI:** 10.2807/1560-7917.ES.2023.28.1.2200937

**Published:** 2023-01-05

**Authors:** Charlotte Munkstrup, Frederikke Kristensen Lomholt, Hanne-Dorthe Emborg, Karina Lauenborg Møller, Jesper Schak Krog, Ramona Trebbien, Lasse S. Vestergaard

**Affiliations:** 1 Infectious Disease Epidemiology and Prevention, Statens Serum Institut, Copenhagen, Denmark; 2 Division of Infectious Disease Preparedness, Statens Serum Institut, Copenhagen, Denmark; 3 Department of Virus and Microbiological Special Diagnostics, Statens Serum Institut, Copenhagen, Denmark

**Keywords:** epidemiology, surveillance, respiratory syncytial virus, RSV, COVID-19, SARS-CoV-2

## Abstract

In the autumn of 2022, Denmark witnessed the second out-of-season epidemic of respiratory syncytial virus (RSV) following widespread societal preventive measures implemented against the coronavirus disease (COVID-19) pandemic during 2020 and 2021. Admissions peaked at twice the level of pre-pandemic seasons. Especially infants below 6 months of age were affected, but also adults over 45 years of age. The current epidemic is dominated by RSV subtype B, unlike the major RSV summer epidemic in 2021 dominated by RSV subtype A.

In March 2020, Denmark introduced a wide range of interventions and measures to prevent the spread of severe acute respiratory syndrome coronavirus 2 (SARS-CoV-2) and mitigate the impact of the COVID-19 pandemic. The measures not only reduced transmission of SARS-CoV-2, but also prevented the spread of other respiratory infections during the remainder of 2020 and during the 2020/21 winter season, such as influenza [1] and respiratory syncytial virus (RSV) [2]. During the summer of 2021, after gradual lifting of COVID-19 measures, RSV presented as a highly unusual out-of-season epidemic in Denmark [3] and in several other countries [4-7]. Measures were reinstalled in late 2021 to prevent the surge of SARS-CoV-2 Omicron variant, but after all measures were ultimately lifted in February 2022, other respiratory infectious diseases returned rapidly, such as influenza [8].

In the autumn and winter season of 2022/23, Denmark experienced again an unusual out-of-season epidemic of RSV. We here present preliminary Danish RSV surveillance data for this season.

## Respiratory syncytial virus surveillance in Denmark

RSV testing is mainly done at hospital level as RSV testing in primary healthcare settings is not recommended. In some cases, testing will be done for the purpose of acute diagnostics, especially among young infants under 6 months of age including those prematurely born, as for them RSV may rapidly lead to severe and complicated illness requiring mechanical ventilation and other intensive care. For adults, testing is typically performed in emergency medical wards as part of examination of patients presenting with signs and symptoms of upper or lower respiratory tract infections, often combined with testing for influenza virus and/or SARS-CoV-2 at the same time.

Mainly PCR is used for testing at the local hospital level and test results are stored in the national Microbiology Database (MiBa). In addition, samples collected as part of sentinel surveillance at general practitioner level and a random subset of RSV-positive samples from the hospital clinical microbiological diagnostic laboratories are forwarded to the national virological reference laboratory at Statens Serum Institut (SSI) in Copenhagen, for testing and subtyping. We retrieved all RSV clinical test results from MiBa [10], RSV test results from the sentinel surveillance, and results from subtyping analysis at SSI, to understand the virological pattern of RSV transmission across Denmark in the current RSV epidemic.

### Definition of respiratory syncytial virus cases and admissions

A case of RSV was defined as any person who had a first positive RSV test in the 2022/23 season. The case definition is based on testing alone and does not consider clinical data. Cases were linked to the Danish National Patient Registry to retrieve information on hospital admissions [11].

An RSV admission is defined as a person admitted to hospital for at least 12 hours where a positive sample for RSV was taken during 4 days before admission or during admission.

An RSV surveillance season lasts from week 21 to week 20 in the following year.

## Out-of-season respiratory syncytial virus epidemic August to December 2022

### Cases

**Figure 1:**
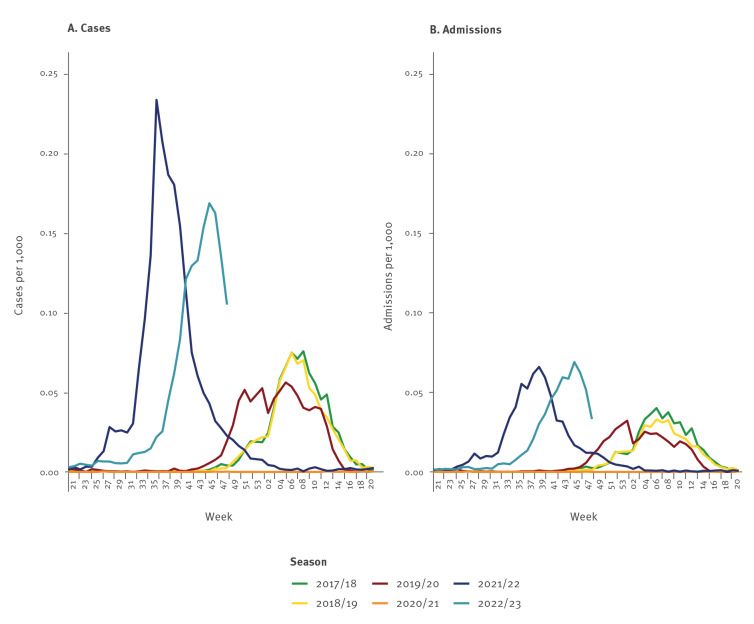
Respiratory syncytial virus (RSV) incidence rates for (A) cases and (B) admissions by week, Denmark, seasons 2017/18 to 2022/23

Up to and including week 48, there were a total of 8,461 cases with a median age of 16 months (interquartile range (IQR): 4 months–37 years) of whom 51.4% (n = 4,348) were males and 48.6% (n = 4,113) females. From around mid-August (week 31) onwards, a gradually increasing number of RSV-positive cases were recorded (Figure 1A). From late-August (week 34), the number of registered RSV cases increased steeply and seems to have peaked in week 45 with a weekly incidence rate of 0.168 per 1,000 population.

For the three pre-COVID-19 pandemic seasons 2017/18 to 2019/20, the average peak incidence rate was 0.067 per 1,000 population, meaning that the incidence rate in the current epidemic is about 2.5 times higher than in the pre-COVID-19 seasons. However, the peak in the 2022/23 season was lower than in the summer epidemic of 2020/21.

### Admissions

Up to and including week 48, there were a total of 3,417 persons admitted with a median age of 14 months (IQR: 2 months–62 years) of whom 52.4% (n = 1,789) were males and 47.6% (n = 1,628) females. From around mid-August (week 31) onwards, a gradually increasing number of RSV admissions was recorded (Figure 1B), and a steep increase began in late-August (week 34).

The admission incidence rate peaked in week 45 at a level similar to the summer epidemic of 2021. The average weekly peak admission incidence rate for the three pre-COVID-19 seasons was 0.035 per 1,000 population and the peak in the current 2022/23 season was 0.069 per 1,000 population thus far, meaning that the weekly admission incidence rate is twice as high as during the pre-COVID-19 seasons.

**Figure 2:**
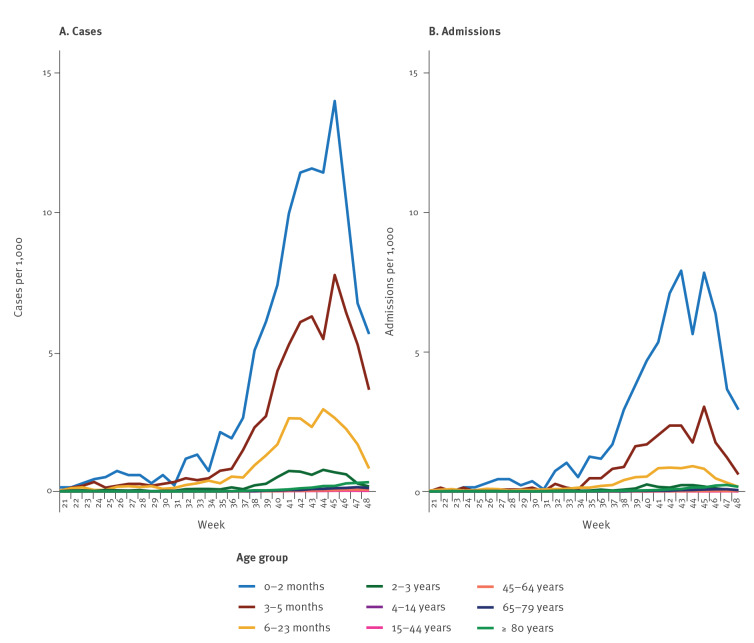
Respiratory syncytial virus (RSV) age dependent incidence rates for (A) cases and (B) admissions by week, Denmark, season 2022/23

**Figure 3:**
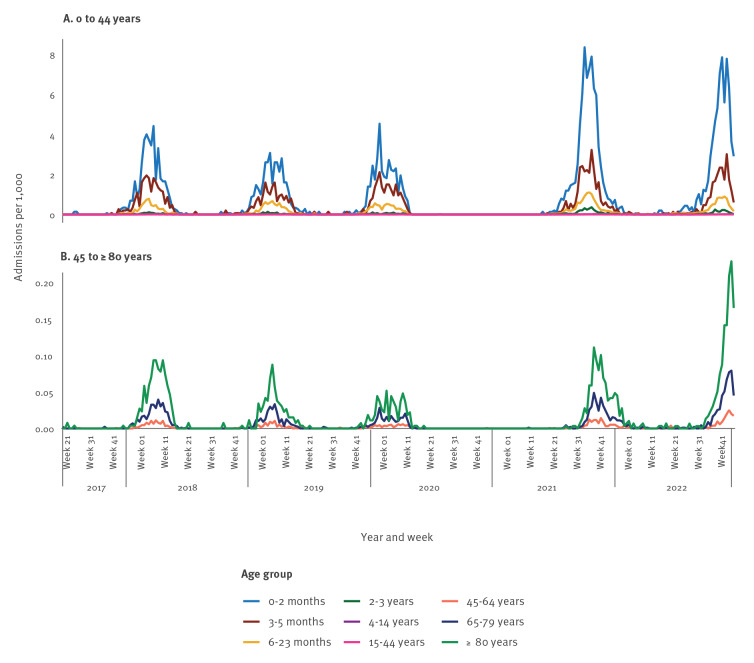
Respiratory syncytial virus (RSV) age dependent admission incidence rate by week in Denmark from 2017 to 2022

There was a clear age-dependent pattern of admissions, with especially infants younger than 6 months of age and children under the age of 2 years being much more likely to be admitted with RSV (Figure 2B) than any other age group. There is, however, also a notable increase in admission incidence rates among all age groups above 45 years old, especially among those aged 80 years and above (see Figure 3B). A total of 107 of 1,026 (10.4%) adults over 45 years old were registered to have received intensive care during their admission.

Please note the different scaling of the y-axis in panel A and B.

### Respiratory syncytial virus testing

In 2021, an increase in year-round testing for RSV was established and increased even more in 2022. The weekly testing rate in the current epidemic resembles the test activity seen in previous seasonal peaks with weekly testing rates peaking at respectively 0.51, 0.63 and 0.59 per 1,000 population in the pre-pandemic seasons 2017/18, 2018/19 and 2019/20 and this season’s peak testing rate at 0.60 per 1,000 population in week 47.

### Respiratory syncytial virus subtypes

In this 2022/23 season, RSV-B was the primary subtype circulating, accounting for 98% (n = 1,927) of the subtyped samples (n = 1,967). Only sporadic cases of RSV-A (1.7%, n = 34) were observed and even fewer had both RSV-A and RSV-B (0.3%, n = 6). In the previous season of 2021/22, RSV-A was the dominating subtype in Denmark, but it ended with a tail of RSV-B (data not shown).

## Discussion

In Denmark, before the COVID-19 pandemic, RSV usually circulated primarily during the winter months every year, with a fluctuating onset in the early winter, between weeks 46 and 52 [12].

When the COVID-19 pandemic emerged in 2020, against which multiple societal restrictions and preventive measures were implemented, a marked decrease in RSV transmission was rapidly noted, followed by an almost complete absence of RSV transmission throughout the following 2020/21 winter season. Since then, Denmark experienced two large atypical out-of-season epidemics: a summer epidemic in 2021 and an autumn epidemic in 2022. In these two epidemics, there were both more cases and more RSV hospital admissions than during the typical winter peaks. Importantly, this cannot be explained by an increased RSV testing activity, as the current testing level corresponds to pre-pandemic RSV seasonal peaks.

The summer epidemic of 2021, atypical in timing and intensity, was most likely facilitated by immunity debt due to absent circulation of RSV while measures against COVID-19 were in place [13] and the epidemic coincided with the gradual lifting of restrictions. The size of the current epidemic might indicate that lack of immunity is still an important factor, but other factors such as the circulating RSV subtypes and cross-immunity between them may also play a role.

Although this epidemic is atypical, it seems to have a typical age-related RSV risk pattern [14] with especially infants and younger children being affected. However, there is a notable increase in incidence rate of both cases and admissions in older adults, especially in the age group 80 years and above. With RSV acute respiratory infection constituting substantial disease burden in older adults [15], this is an important signal which will need further attention and evaluation.

Following the COVID-19 pandemic, there has been concern of double- or even triple epidemics with concurrent COVID-19, influenza and RSV. Indeed, this is a possible scenario given the uncertain development of the current epidemic of RSV, which is still ongoing at a high level especially among elderly people over 80 years of age. This is not only relevant in Denmark, but also internationally as many countries are experiencing an RSV epidemic currently [16].

As an important limitation of this report, the hospital admission data presented do not consider whether RSV is the main cause for admission. It is also important to note that the further evolvement of the epidemic is still unknown at this stage.

## Conclusions

After two consecutive large out-of-season RSV epidemics, the obvious question is: When will we be back to ‘normal’ epidemic patterns, both in timing and intensity? The current epidemic moving closer in time to the winter months suggests that there may be a gradual approaching towards usual RSV seasonality like before the COVID-19 pandemic. The two recent atypical epidemics emphasise, however, the importance of year-round real-time RSV surveillance with both laboratory and epidemiological data, as also recommended by the European Centre for Disease Prevention and Control [17], since it is hard to predict when the next RSV epidemic will emerge and what will characterise it in terms of size, subtype circulation and age distribution. There is a need for special attention to the impact of RSV among elderly people, which are highly vulnerable in case of a triple epidemic.
