# Erratum for Euro Surveill. 2023;28(1)

**DOI:** 10.2807/1560-7917.ES.2023.28.5.230202e

**Published:** 2023-02-02

**Authors:** Eurosurveillance editorial team

**Affiliations:** 1 European Centre for Disease Prevention and Control (ECDC), Stockholm, Sweden

In the article ‘Early and intense epidemic of respiratory syncytial virus (RSV) in Denmark, August to December 2022’ by Munkstrup et al., a publication error caused some of the citations to be incorrectly numbered. This was corrected on 26 January 2023, and we apologise for any inconvenience this error may have caused.

